# Accuracy of Anorectal Manometry to Detect the Rectoanal Inhibitory Reflex in Children: Awake Versus Under General Anesthesia

**DOI:** 10.1097/MPG.0000000000003779

**Published:** 2023-04-10

**Authors:** Desiree F. Baaleman, Samir Mishra, Ilan J.N. Koppen, Jac. M. Oors, Marc A. Benninga, Neetu Bali, Karla H. Vaz, Desale Yacob, Carlo Di Lorenzo, Peter L. Lu

**Affiliations:** From the * Division of Gastroenterology, Hepatology, and Nutrition, Department of Pediatrics, Nationwide Children’s Hospital, Columbus, OH, USA; the † Department of Pediatric Gastroenterology and Nutrition, Emma Children’s Hospital, Amsterdam UMC, University of Amsterdam, Amsterdam, the Netherlands; the ‡ Amsterdam UMC, Univ of Amsterdam, Gastroenterology and Hepatology, Amsterdam Gastroenterology Endocrinology Metabolism, Meibergdreef 9, Amsterdam, the Netherlands.

**Keywords:** anesthesia, anorectal manometry, children, constipation, Hirschsprung disease

## Abstract

Our objective was to investigate if there is a difference in the detection of the rectoanal inhibitory reflex (RAIR) when an anorectal manometry (ARM) is performed awake or under general anesthesia.

**Methods:**

A retrospective review of ARM studies was performed to identify children who had undergone ARMs both while awake and under general anesthesia. We compared ARM outcomes including the detection of the RAIR and anal canal resting pressure.

**Results:**

Thirty-four children had received ARMs both while awake and under general anesthesia (53% female, median age at first ARM 7.5 years [range 3–18 years]). In 9 of 34 (26%) children the RAIR was solely identified during ARM under general anesthesia and not during ARM while awake. In 6 of 9 (66%) this was unrelated to the balloon volumes used during balloon inflations. In 4 of 34 (12%) children, assessment of the RAIR was inconclusive during ARM under general anesthesia due to too low, or loss of anal canal pressure. In 2 of those children, ARMs while awake showed presence of a RAIR. Anal canal resting pressures were higher during ARM while awake versus ARM under general anesthesia (median 70 [interquartile range, IQR 59–85] vs 46 mmHg [IQR 36–65] respectively, *P* < 0.001).

**Conclusions:**

General anesthesia may affect the detection of a RAIR in 2 ways. On the one hand, it may facilitate better visualization in children in whom a RAIR could not be visualized while awake. On the other hand, it may cause a loss of anal canal pressure resulting in an inconclusive test result.

## Graphical abstract

**Figure:**
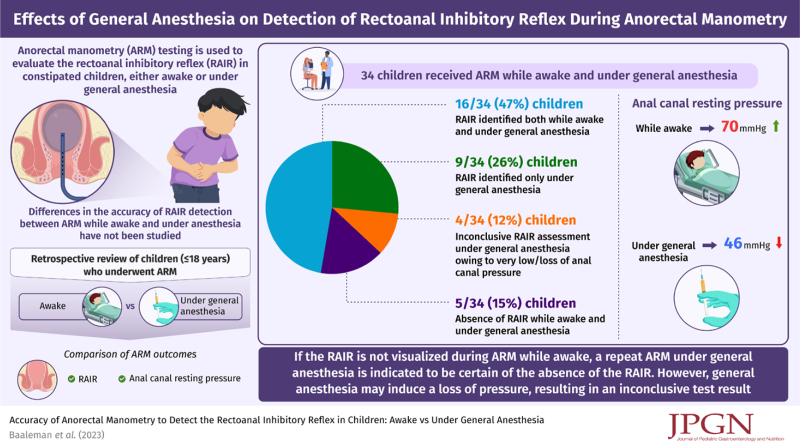

**What Is Known**

- Studies on the effect of general anesthesia on anorectal manometry (ARM) show it decreases anal canal resting pressure.
- Differences in the accuracy of the detection of the rectoanal inhibitory reflex (RAIR) between ARM while awake or under general anesthesia have not been thoroughly investigated.

**What Is New**

- General anesthesia may facilitate better visualization of the RAIR in children in whom the RAIR was not visualized while awake regardless of balloon volumes used.
- General anesthesia may induce a loss of pressure resulting in an inconclusive test result.
- If the RAIR is not visualized during ARM while awake, a repeat ARM under general anesthesia is indicated to be certain of the absence of the RAIR.

In children with severe constipation, anorectal manometry (ARM) testing may be performed to evaluate the neuromuscular function of the anus and rectum (1). It is the most commonly performed motility test in children and provides insight in anal sphincter function, defecation dynamics, rectal sensation, and the presence or absence of the rectoanal inhibitory reflex (RAIR) (2). The RAIR is an involuntary anal reflex mediated by a complex intramural neuronal plexus that results in a decrease of the internal anal sphincter (IAS) pressure following distension of the rectum. Such distension can be caused by gas, feces, or an inflated balloon during ARM testing (3). In patients with Hirschsprung disease, the RAIR is absent due to an abnormal development of the enteric nervous system resulting in the absence of ganglion cells (4). Additionally, the RAIR is occasionally found to be absent in children with normal presence of ganglion cells who are then diagnosed with internal anal sphincter achalasia (IASA) (5,6). The clinical significance of this diagnosis is still unclear (5). ARM is preferably performed awake to assess rectal sensation, squeeze pressure, and defecation dynamics (2,7). However, if a child is unable to tolerate an ARM awake or if the child has other procedures requiring sedation (eg, anal sphincter botulinum toxin injection), the ARM may be performed under general anesthesia. In a previous study, we identified children in whom the RAIR was only identified during ARM under general anesthesia and not during ARM while awake which led to the design of the current study (5). Our primary objective is to investigate if there is a difference in the accuracy of an ARM to detect the RAIR when an ARM is performed awake compared to when an ARM is performed under general anesthesia. Our secondary objective is to evaluate effects of anesthetic drugs on anal canal resting pressure and the detection of the RAIR.

## METHODS

We performed a retrospective review of all children ≤18 years of age who had more than 1 ARM study performed at our institution between 2012 and 2020. The local Institutional Review Board approved the study protocol (STUDY00000294). We identified all children with ARM studies performed both awake and under general anesthesia. We excluded children in whom studies were performed more than 2 years apart to minimize effects of age or time. If possible, we also included children diagnosed with Hirschsprung disease to serve as positive controls. We recorded demographic information, medical history, surgical history, anesthetic drugs used, and ARM outcomes. In order to adequately interpret these findings, we extended our discussion with a narrative review of the current literature on anal sphincter physiology and effect of anesthetics. The ARM was performed in accordance with current guidelines; details on the procedure and the assessment are provided in Supplemental Digital Content 1, http://links.lww.com/MPG/D117.

### ARM Outcomes

In the current literature, there is no consensus on the appropriate cut-off to consider the RAIR present. Cut-offs described in consensus documents range from a 5 mmHg decrease to a 25% pressure drop (2,8). For this study, the RAIR was considered to be present when a drop of >15% in anal canal pressure was observed during balloon inflation (9). We considered a decrease of 5 mmHg too loose, as it is difficult to differentiate such a small change from movement artifacts and the change may easily be fabricated by movement of the measuring indicator during analysis. A drop of 25% seemed too strict, especially in children who may contract their external anal sphincter during the measurement which may impair visualization of the relaxation. Children were diagnosed with IASA if they had an absent RAIR during ARM awake and under general anesthesia and a normal rectal biopsy. If a reliable measurement of the RAIR was not possible, for example, due to extremely low pressures, the RAIR was considered inconclusive. The anal canal resting pressure was calculated as the mean pressure during a resting period of at least 30 seconds (2). We compared anal canal resting pressure between the first and second ARM to evaluate for a possible confounding effect of time/aging of the child. We then investigated the effects of (different types of) general anesthesia on anal canal resting pressure.

### Statistical Analyses

Statistical analyses were conducted with SPSS for Windows, version 24 (SPSS, Inc., Chicago, IL). Data are presented as medians and interquartile ranges (IQRs). Differences in anal canal resting pressures within individuals were compared using the Wilcoxon signed-rank test. In order to minimize the effect of inter-individual differences in anal canal resting pressures when analyzing effect of anesthetic drugs, we calculated the *delta* resting pressure (Δ resting pressure); the difference between anal canal resting pressures while awake and under general anesthesia within each individual. We used this variable to examine the different effects of the individual anesthetic drugs on anal canal resting pressure using the Mann-Whitney *U* test. Since many children used a combination of anesthetic drugs, children were divided into subgroups of children who received the same combination of drugs (eg, propofol and sevoflurane) and we compared Δ resting pressure between subgroups using the Kruskal-Wallis test. A *P* value of <0.05 was considered statistically significant.

## RESULTS

We reviewed charts of 91 patients who had undergone at least 2 ARM studies and identified 34 children who had the study performed both awake and under general anesthesia (53% female, median age at first ARM 7.5 years [IQR 5.9–9.1 years, range 3–18 years]), see Supplemental Digital Content 2, http://links.lww.com/MPG/D118. Prior to the ARM, most children had a diagnosis of functional constipation (n = 33; 97%); one patient was diagnosed with Hirschsprung disease. Fourteen children (41%) had their first ARM performed awake, and all 14 had it repeated under general anesthesia due to absence or uncertainty about the presence of the RAIR (Fig. 1). The 3 children with a present RAIR during the first awake ARM were considered incomplete during assessment at time of their ARM. During repeat assessment during this study, these RAIRs were considered present. Twenty children (59%) had their first ARM performed under general anesthesia and had it repeated while awake to evaluate for pelvic floor dyssynergia (n = 17) or because their anal canal pressure during ARM was too low to evaluate for the RAIR (n = 3). Median time between ARMs was 2.5 months (IQR 0.0–11.5 months).

**Figure:**
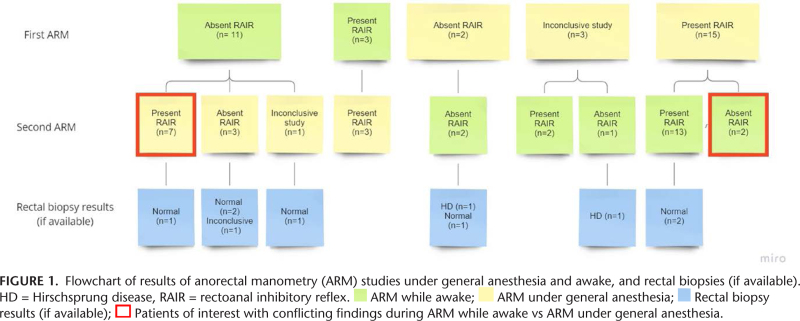


### Rectoanal Inhibitory Reflex

In 9 of 34 (26%) children, the RAIR was solely identified during ARM performed under general anesthesia and not while awake (Table 1). Time between ARMs in these children ranged from 0 to 9 months. In 3 of 9 (33%) children, the maximum balloon inflations during ARM while awake were low, with volumes of 10, 20, and 30 mL, respectively. No further balloon inflations had been done in these children due to severe discomfort and distress. In these children, ARM under general anesthesia showed presence of the RAIR at volumes of 20, 30 and 60 mL, respectively. In 5 of 9 (56%) children, maximum balloon volumes during ARM while awake ranged between 80 and 250 mL. These same children had an identifiable RAIR at lower volumes ranging between 10 and 60 mL during ARM under general anesthesia. In one patient, several balloon inflations were performed during ARM while awake, but volumes were not recorded. In this patient, the RAIR was identified with the first balloon inflation of 10 mL when the study was repeated under general anesthesia.

**TABLE 1.** Comparison of presence of the rectoanal inhibitory reflex (RAIR) during anorectal manometry while awake and under general anesthesia (N = 34)

RAIR awake	RAIR under general anesthesia		
	Present	Absent	Inconclusive
Present	16 (47%)	0 (0%)	2 (6%)
Absent	9 (26%)	5 (15%)	2 (6%)

In 4 of 34 (12%) children, the RAIR was inconclusive when the ARM was done under general anesthesia due to a very low anal canal resting pressure or due to complete loss of pressure. Initial resting pressures in these children ranged from 13 to 86 mmHg. In these same children, ARM while awake showed normal anal canal resting pressures in all of them, the presence of a RAIR in 2 children and absence of a RAIR in the other 2 children. One of these children with an absent RAIR during ARM while awake, and the highest baseline pressure of 86 mmHg during ARM under general anesthesia, was diagnosed with Hirschsprung disease via rectal biopsy. All 4 children were induced with propofol when the ARM was done under general anesthesia (doses were 1.87, 3.85, 3.98, and 5.67 mg/kg, respectively). During the complete procedure, 1 patient received only propofol; 1 patient received propofol and sevoflurane; 1 patient received propofol, sevoflurane, and nitrous oxide; and 1 patient received propofol, sevoflurane, nitrous oxide, and fentanyl.

In 5 of 34 (15%) children, the RAIR was absent during ARM awake and ARM under general anesthesia (Fig. 1). All underwent rectal biopsy; one of these children was diagnosed with Hirschsprung disease. Resting pressures of this patient were not elevated; 45 mmHg during ARM while awake and 37 mmHg during ARM under general anesthesia.

### Anal Canal Resting Pressure

In order to evaluate temporal effects, we compared anal canal resting pressures between the first and second ARM, which showed no significant difference (median 60 [IQR 40–74] vs 67 [IQR 45–77] mmHg respectively, *P* = 0.278). Anal canal resting pressures were higher during ARM while awake versus ARM under general anesthesia (median 70 [IQR 59–85] vs 46 [IQR 36–65] respectively, *P* < 0.001) (Table 2). Anesthetic drugs utilized during ARM under general anesthesia included (a combination of) sevoflurane (n = 32; 94%), propofol (n = 26; 76%), nitrous oxide (n = 19; 55%), fentanyl (n = 11, 32%), dexmedetomidine (n = 1; 3%), and rocuronium (n = 1; 3%). Children who received sevoflurane or propofol during ARM under general anesthesia had lower anal canal resting pressures under general anesthesia compared to while being awake (median change in resting pressure 20.5 mmHg [IQR 2–38, *P* < 0.001] and 29.5 [IQR 9–34, *P* < 0.001], respectively). There was no significant difference in change in resting pressures among anesthesia subgroups (*P* = 0.163).

**TABLE 2.** Anal canal resting pressure per study condition and per anesthetics used

	N	Pressure in mmHg, median (IQR)	Change in pressure, *median (IQR)	*P* value compared to awake
Awake	34	70 (59–85)	n/a	n/a
Under general anesthesia	34	46 (36–65)	21.5 (3 to 40)	**<0.001**
Sevoflurane	32	46 (37–66)	20.5 (2 to 38)	**<0.001**
Propofol	26	42 (31–60)	29.5 (9 to 45)	**<0.001**
Induction with propofol	20	42 (36–63)	29.5 (6 to 46)	**0.001**
Nitrous oxide	19	53 (38–68)	8 (–2 to 20)	0.080
Fentanyl	11	42 (39–56)	20 (1 to 32)	0.062
Anesthesia with propofol and sevoflurane	9	40 (20–59)	46 (22 to 55)	
Anesthesia with propofol, sevoflurane, and nitrous oxide	6	57 (36–78)	4 (–11.5 to 30)	
Anesthesia with propofol, sevoflurane, nitrous oxide, and fentanyl	6	42 (31–47)	25.5 (–0.6 to 34)	
Anesthesia with sevoflurane and nitrous oxide	5	54 (53–69)	6 (1 to 9)	
Anesthesia with propofol, sevoflurane, and fentanyl	2	50 (40–60)	21.5 (3 to 40)	
Anesthesia with sevoflurane, nitrous oxide, and fentanyl	2	53 (37–68)	–13.5 (–35 to 8)	
Anesthesia with propofol only	2	39 (25–53)	40.5 (36 to 45)	
Anesthesia with sevoflurane and fentanyl	1	51	34	
Anesthesia with propofol, sevoflurane, nitrous oxide, dexmedetomidine, and rocuronium	1	56	21	

* Anal canal resting pressure awake minus anal canal resting pressure under general anesthesia. Bold *P* values denote statistical significance at the *P* < 0.05 level.

### Diagnoses and Changes in Treatment after ARM

Among all 34 children, 14 (41%) had normal ARM findings, 13 (38%) were diagnosed with pelvic floor dyssynergia, 3 (9%) were diagnosed with IASA, 2 (6%) were diagnosed with IASA and pelvic floor dyssynergia, and 2 (6%) were diagnosed with Hirschsprung disease. Children who had an absent RAIR during ARM while awake were scheduled for a repeat ARM under general anesthesia and subsequent botulinum toxin injection after the second ARM. Of the 14 children with normal ARM findings, 5 (36%) received anal sphincter botulinum toxin injections, 1 (7%) started antegrade continence enemas, 1 (7%) received a sacral nerve stimulator, and for the remaining 7 (41%) treatment did not change. The majority of the 13 children with pelvic floor dyssynergia started with biofeedback training (n = 8; 62%) and in some children this was combined with anal sphincter botulinum toxin injections (n = 3; 23%). Two children (15%) received anal sphincter botulinum toxin injections alone and for 3 (23%) treatment did not change. All children with IASA received anal sphincter botulinum toxin injections (n = 5; 100%), and the 2 IASA children with pelvic floor dyssynergia additionally started with biofeedback training. The two children with Hirschsprung disease were surgically treated.

## DISCUSSION

In this study we found that in a quarter of children with constipation who had undergone ARM both awake and under general anesthesia, the RAIR was only identified during ARM under general anesthesia. This appeared to be unrelated to the balloon volumes used during the ARMs in 6 of these children. In 4 children the RAIR was inconclusive during ARM under general anesthesia due to too low, or loss of anal canal pressure. During ARM while awake, the RAIR was absent in 2 of these children, and present in the other 2 children. Average anal canal resting pressure was significantly lower during ARM under general anesthesia compared to ARM while awake. These findings suggest that the detectability of the RAIR may be affected by either the level of consciousness of the patient and/or anesthetic drugs. In order to put these findings into perspective, it is important to understand the normal tone generation and innervation of the internal and external anal sphincter and how they may be affected by anesthetic drugs.

### Anal Canal Pressure and Innervation

The IAS is made of smooth muscle derived from the circular muscle layer of the rectum, forming a ring that, if contracted, encloses the anal canal circumferentially in a spiral orientation and contributes to the majority of the anal pressure at rest (70%–85%) (10,11). The IAS is innervated by both excitatory and inhibitory motor neurons which receive neural input from intramural plexuses and autonomic nerves in the lesser pelvis (12). Tone generation in the IAS likely occurs as a direct result from smooth muscle cells and waves generated by the interstitial cells of Cajal (13). Activity of the IAS is considered to be modulated by autonomic innervation, where sympathetic nerves act excitatory and parasympathetic nerves act inhibitory (3). Whereas the tonic activity of the IAS is mostly myogenic, the relaxation of the IAS is a neurogenic reflex, mediated by non-adrenergic non-cholinergic nerves that release nitric oxide, vasoactive intestinal polypeptide, and possibly carbon monoxide (13). In contrast to the IAS, the external anal sphincter and puborectalis muscle are composed of striated muscle under spinal and cortical control (14). Although their contribution to anal canal resting pressure is minimal, both are able to contract voluntarily and involuntarily to maintain continence (15,16). Their voluntary contractions are regulated by the pudendal nerve which originates from the second to fourth sacral roots (10,17). They seem to mainly play a role in sudden straining, therefore their contraction may play a role during sudden inflation of the rectal balloon during ARM (18,19).

### General Anesthesia and the Anal Sphincters

A previous study has shown that contraction of the external anal sphincter in response to relaxation of the IAS also occurs during normal sleep (20). This contraction is thought to counteract IAS relaxation and helps to maintain pressure in the anal canal. Anesthetics may hinder this reflex contraction thereby decreasing anal canal pressure, which may explain why in some children we only observed a RAIR during ARM under general anesthesia. A study which recorded 20–24 hours of anorectal activity in 10 healthy adults found that the anal canal pressure was significantly lower, around 45%, when subjects were asleep compared to when they were awake (21). The unconsciousness of the patient under general anesthesia may therefore explain the majority of the decrease in anal canal resting pressure. Our findings are in line with a recent study which evaluated the effect of propofol on ARM outcomes in 27 children (22). Although the authors state that propofol did not affect the presence of the RAIR, they describe that in 2 children they were only able to show the RAIR under general anesthesia. They attribute this to the uncooperativeness of the children without providing details on balloon volumes used. Moreover, they do not specify their definition of a present RAIR. In contrast to our study, the authors do not describe a loss in anal canal pressure during ARM under general anesthesia resulting in the inability to measure the RAIR. The loss of anal canal pressure during ARM under anesthesia may be secondary to the combination of: (1) a lower basal resting pressure during sleep (21); (2) the effect of anesthetics on the muscle activity of both the internal and external anal sphincter (23); and (3) by the hindrance of anesthetics on the reflex contraction of the external anal sphincter (22). Given the many combinations of anesthetic drugs used in our study population, it is difficult to draw any firm conclusions on individual effects of different anesthetic drugs on anal canal resting pressure or their effect on the RAIR. Findings of previous studies investigating the effect of anesthetics on anorectal motility are summarized in Table 3 (22–28). Comparative data of mono-therapeutic studies or randomized controlled trials are needed to investigate effects of specific anesthetics on ARM findings. During these studies the doses and depth of anesthesia should be taken into account. Other factors that should be taken into consideration when determining which (combination of) anesthetic drug(s) is preferred include provider/patient preference, time to spontaneous awakening (29), adverse reactions (30), and costs.

**TABLE 3.** Anesthetic agents, their mechanism of action, and possible effects on anorectal motility

Anesthetic agent	Mechanism of action	Effect on anorectal motility
Atropine	A competitive antagonist of the muscarinic acetylcholine receptor.	No effect on the presence or absence of the RAIR (24)
Glycopyrrolate	A competitive antagonist of the muscarinic acetylcholine receptor.	May have an inhibitory effect on either the sensory or motor aspects of the RAIR (24)
Ketamine	A phenylpiperidine derivative with a complex mechanism of action resulting in anesthesia and analgesia.	No effect on anal canal resting pressure of the presence or absence of the RAIR (25,26)
Midazolam	A benzodiazepine, including sleep induction, sedation, anxiolysis, and amnesia.	No effect on the presence or absence of RAIR (24)
Propofol	Nonopioid, nonbarbiturate intravenous sedative hypnotic by positive modulation of the inhibitory function of the neurotransmitter g-aminobutyric acid (GABA) through GABA-A receptors	Lowers anal canal resting pressure (22,23,27,28)
		May increase percentage of internal anal sphincter relaxation after balloon distension (22)
Sevoflurane	A positive allosteric modulator of the GABA-A receptor, an NMDA receptor antagonist, potentiates glycine receptor currents, and inhibits nAChR and 5-HT3 receptor currents.	Lowers internal anal sphincter amplitude compared to sedation with propofol (23)

### Clinical Consequences

It is not completely clear why in a subset of children the RAIR was only visualized during ARM under general anesthesia. This may be the result of a decrease in pressure during ARM under general anesthesia or the hindrance of the reflex contraction of the external sphincter by anesthetics, as discussed in the previous section. However, if the increase in external sphincter pressure during ARM while awake prevents the visualization of the RAIR, we would expect to encounter this in many more children who undergo ARM while awake. Examination-related technical issues seem unlikely to explain this finding as no air leakage or catheter displacement was observed, and rectal balloon volumes should have been sufficient to provide reliable measurements. Anxiety of the patient may increase movement artifacts or hinder adequate balloon inflation but, if the procedure can be executed accurately, this should not influence the presence of the involuntary RAIR. Incorrect identification of an absent RAIR may result in the unnecessary performance of rectal biopsies and the incorrect diagnosis of IASA. However, although children with an absent RAIR while awake and a present RAIR under general anesthesia may not be diagnosed with IASA, their symptoms might not differ much from children diagnosed with IASA. In these children with only a visible RAIR under general anesthesia, it is likely that the RAIR does occur while they are awake, but is disguised by other anal canal pressures that make it unrecognizable during ARM while awake. Still, the ARM while awake more likely represents what they experience when they try to defecate during the day. Therefore, one could argue that these children may experience obstructive symptoms in daily life similar to a child diagnosed with IASA. These findings may have a major impact on the way in which IASA is currently diagnosed. In our sample, 9 children would have been misdiagnosed with IASA if they would not have undergone a repeat ARM (5).

Limitations of our study are inherent to its retrospective design. Not all ARMs were performed on the same day and various anesthetics were used. Because there is no consensus on the definition of a present RAIR, we used a 15% drop in pressure as cut-off to consider the RAIR present (9). If the RAIR was defined another way, that would of course change our results. We only evaluated children from 3 years of age which limits the generalizability of our findings. We did not collect other clinical data or our population, such as the presence of a megarectum. Changes in rectum size may affect the ability to detect a RAIR. However, we believe this is unlikely to happen within a short period of time, as the time between the 2 ARMs in children with a RAIR present only during awake ARM was 9 months at maximum. Future studies may evaluate the effects of repeating an ARM in children currently diagnosed with IASA, as well as comparing long-term clinical outcomes of children who only had a visible RAIR under general anesthesia with children diagnosed with IASA. In addition, midazolam may be used in children who are anxious for the ARM, which may limit the need to repeat the ARM under general anesthesia.

### CONCLUSIONS

The effect of general anesthesia on the detection of the RAIR is two fold. General anesthesia may facilitate better visualization in children in whom the RAIR could not be visualized while awake but may also induce a loss of pressure resulting in an inconclusive test result. If the RAIR is not visualized during ARM while awake, our data suggest that a repeat ARM under general anesthesia is indicated regardless of the size of balloon volumes used during the awake study when there is a clinical need to be certain of the absence of the RAIR. However, the clinical significance of the absence of the RAIR during ARM while awake, as well as the diagnosis of IASA, remains unclear and more research is indicated to adequately interpret these abnormal findings.

## Acknowledgments

We would like to acknowledge the assistance of Sujana Dontukurthy in providing advice in completing the research project. Preliminary findings were presented as poster at the NASPGHAN/APGNN/CPNP 2020 Annual Meeting, as poster of distinction at DDW 2021, and as oral presentation at WCPGHAN 2021.

## Supplementary Material


