# Supplementary material for: Accuracy of Anorectal Manometry to Detect the Rectoanal Inhibitory Reflex in Children: Awake Versus Under General Anesthesia
Source: J Pediatr Gastroenterol Nutr. 2023 Apr 10;76(6):731–6. doi: 10.1097/MPG.0000000000003779 (PMC10171289; doi:10.1097/MPG.0000000000003779)
Supplement: Supplementary file 1 [file mpg-76-731-s001.pdf]

Supplemental Digital Content 1 – details on anorectal manometry procedure and assessment

Anorectal manometry procedure

Indications for ARM testing at our institution included evaluation of the RAIR, anal canal resting pressure, pelvic floor dynamics, and rectal sensation. The ARM was performed with either a high-resolution solid-state catheter (UniTip High Resolution Catheter, model number K12959-L5-1038-D from Unisensor AG) or a water-perfused catheter (Mui Scientific part number SR8B-5-0-0-0-0-0-0-0, Mississauga, Ontario, Canada) and executed in accordance with societal guidelines (2). After insertion of the catheter, all studies began with examination of the resting pressure of the anal sphincter. When a child was awake and very nervous, a more accurate resting pressure measurement was obtained at the end of the study. After measurement of the anal sphincter resting pressure, the presence of the RAIR was evaluated by several rectal balloon inflations with incremental volumes (2, 7). Under general anesthesia the rectal balloon was inflated until the RAIR was visualized, or up to a volume of at least 60 mL or higher (based on age and weight). When awake, the rectal balloon was inflated with incremental balloon volumes until children reported a sensation (generally discomfort or pain). Since increased rectal distension generally results in a higher percentage of IAS relaxation, we examined the RAIR during the three balloon inflations with the largest volumes. If during one of those three balloon inflations the resting pressure was extremely low or the catheter migrated, we used measurements with smaller balloon volumes instead. Patients who were under general anesthesia were in supine position during the procedure, while children who were awake laid on their left side. Additionally, in older children who were awake, squeeze and push (or bear down) maneuvers were performed if possible, and rectal sensory thresholds during rectal balloon inflations were recorded. Five different experienced pediatric gastroenterologist specialized in motility disorders performed the ARMs together with two experienced pediatric motility nurses.

Anorectal manometry assessment

A physician with training in interpreting manometry tests assessed each ARM study for the presence or absence of the RAIR. For studies in which the second assessment differed from the original report by a pediatric gastroenterologist, another pediatric gastroenterologist with advanced training in motility disorders performed a third assessment. All analyses of

manometric data were performed using commercially available software (Solar GI HRM v9.1, Medical Measurement Systems (MMS), Enschede, the Netherlands).
