# Supplementary figures and images for: Accuracy of Anorectal Manometry to Detect the Rectoanal Inhibitory Reflex in Children: Awake Versus Under General Anesthesia
Source: J Pediatr Gastroenterol Nutr. 2023 Apr 10;76(6):731–6. doi: 10.1097/MPG.0000000000003779 (PMC10171289; doi:10.1097/MPG.0000000000003779)

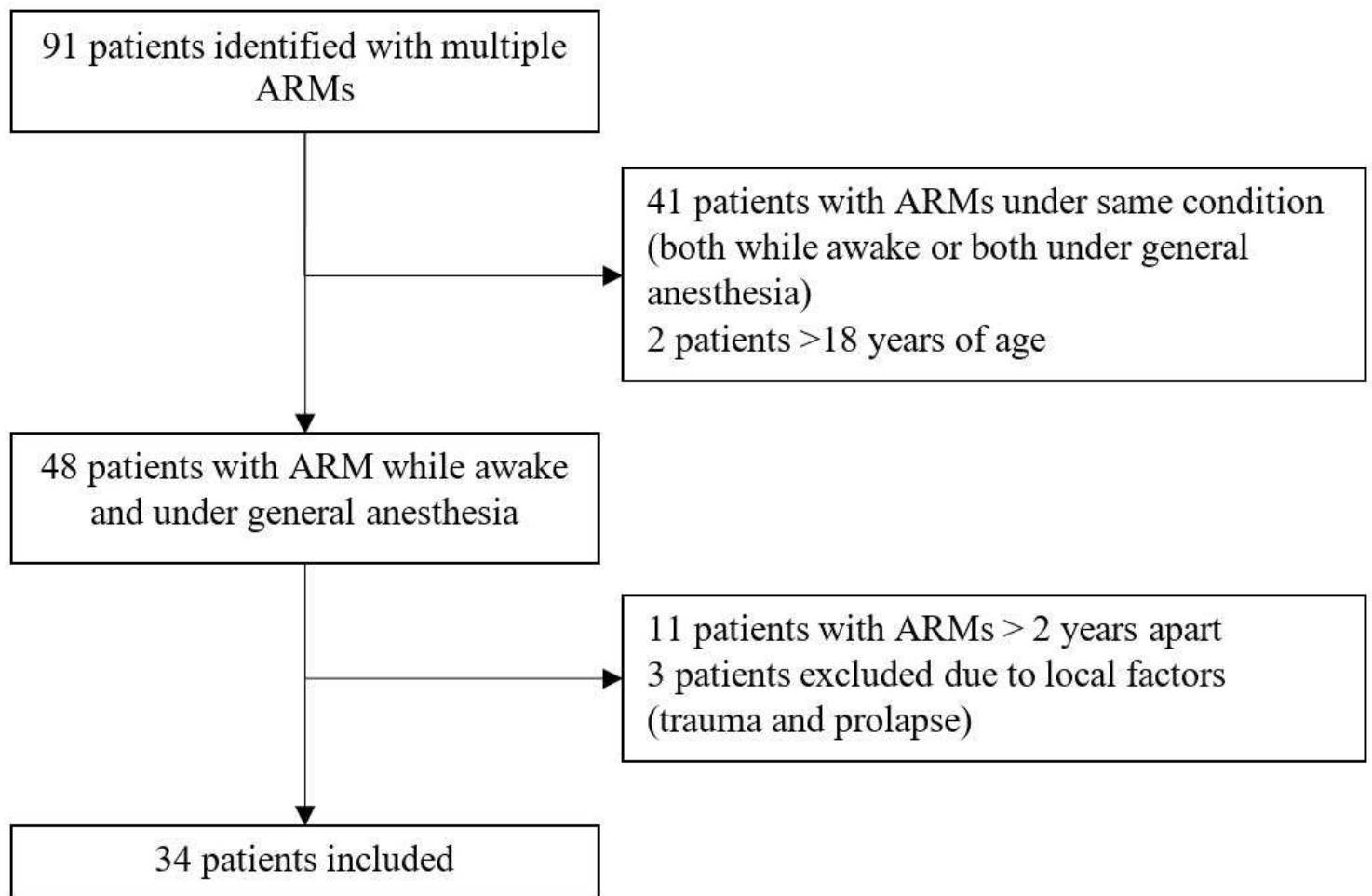

Supplement: Supplementary file 2 [file mpg-76-731-s002.pdf]
